# Preventing illegal seafood trade using machine-learning assisted microbiome analysis

**DOI:** 10.1186/s12915-024-02005-w

**Published:** 2024-09-11

**Authors:** Luca Peruzza, Francesco Cicala, Massimo Milan, Giulia Dalla Rovere, Tomaso Patarnello, Luciano Boffo, Morgan Smits, Silvia Iori, Angelo De Bortoli, Federica Schiavon, Aurelio Zentilin, Piero Fariselli, Barbara Cardazzo, Luca Bargelloni

**Affiliations:** 1 Department of Comparative Biomedicine and Food Science, University of Padova, Viale Dell’Università 16, Legnaro, 35020 Italy; 2 La Vongola Verace Di Chioggia, Chioggia, Italy; 3 LEMAR, UMR 6539 CNRS/UBO/IRD/IFREMER, Institut Universitaire Européen de La Mer, Place Nicolas Copernic, Plouzané, 29280 France; 4 Experteam S.R.L, Via Della Libertà, 12, Marghera, 30175 Italy; 5 Almar Soc. Coop. Agricola Arl, Via G. Raddi, 2, Marano Lagunare, 33050 Italy; 6 Department of Medical Sciences, University of Torino, Via Santena 19, Turin, 10126 Italy

**Keywords:** Machine learning, Food traceability, Microbiota 16S, Manila clam, North Adriatic sea, Illegal unreported unregulated (IUU) fishing

## Abstract

**Background:**

Seafood is increasingly traded worldwide, but its supply chain is particularly prone to frauds. To increase consumer confidence, prevent illegal trade, and provide independent validation for eco-labelling, accurate tools for seafood traceability are needed. Here we show that the use of microbiome profiling (MP) coupled with machine learning (ML) allows precise tracing the origin of Manila clams harvested in areas separated by small geographic distances. The study was designed to represent a real-world scenario. Clams were collected in different seasons across the most important production area in Europe (lagoons along the northern Adriatic coast) to cover the known seasonal variation in microbiome composition for the species. DNA extracted from samples underwent the same depuration process as commercial products (i.e. at least 12 h in open flow systems).

**Results:**

Machine learning-based analysis of microbiome profiles was carried out using two completely independent sets of data (collected at the same locations but in different years), one for training the algorithm, and the other for testing its accuracy and assessing the temporal stability signal. Briefly, gills (GI) and digestive gland (DG) of clams were collected in summer and winter over two different years (i.e. from 2018 to 2020) in one banned area and four farming sites. 16S DNA metabarcoding was performed on clam tissues and the obtained amplicon sequence variants (ASVs) table was used as input for ML MP. The best-predicting performances were obtained using the combined information of GI and DG (consensus analysis), showing a Cohen *K*-score > 0.95 when the target was the classification of samples collected from the banned area and those harvested at farming sites. Classification of the four different farming areas showed slightly lower accuracy with a 0.76 score.

**Conclusions:**

We show here that MP coupled with ML is an effective tool to trace the origin of shellfish products. The tool is extremely robust against seasonal and inter-annual variability, as well as product depuration, and is ready for implementation in routine assessment to prevent the trade of illegally harvested or mislabeled shellfish.

**Supplementary Information:**

The online version contains supplementary material available at 10.1186/s12915-024-02005-w.

## Background

Seafood is crucial for the human diet as it provides an excellent source of high-quality protein, essential omega-3 fatty acids, and various vitamins and minerals. Regular consumption of seafood is linked to numerous health benefits, including heart health, brain function, and overall well-being [1]. Marine bivalves are among the most traded seafood products worldwide [2]. Manila clam (*Ruditapes philippinarum*) is one of the most important mollusc species, with a global production of over four million metric tonnes. In Europe, Italy is the largest producer with ~ 96% of the European production (24,337 t) [3]. Native from South-east Asia (Indo-Pacific), *R. philippinarum* was imported in Europe in the second half of the’70 s and it was introduced in Italy in 1983 [4]. Given its high adaptability, *R. philippinarum* is now commonly found in brackish waters along the northern Adriatic coast especially in the Venice lagoon and nearby areas such as the Po river delta and the lagoon of Marano [3–5].

Despite their high nutritional value, bivalve consumption might pose risks for human health. This is due to their filter-feeding strategy through which these molluscs may accumulate human pathogenic microorganisms as well as metals and/or chemicals present in the water [2, 6, 7]. The European Union (EU) has regulated bivalve harvesting (Regulation (EC) No 853/2004, No 854/2004, No 2073/2005 and No 1021/2008), classifying harvesting areas according to the levels of *Escherichia coli* in the intravalvular liquid per grams of bivalves (EC, 2004a; 2004b, 2005, 2008). Based on these regulations, all species of bivalve molluscs farmed in lagoons must be subjected to depuration processes in order to remove chemical compounds hazardous to humans. Seafood product labels must include the area of provenience (Regulation (EU) No 1379/2013; EU, 2013). Ecolabels have also recently been proposed to improve consumers’ perception and market value of shellfish products [8]. Finally, as bivalves might accumulate significant amounts of chemical pollutants, highly polluted areas might be officially banned for mollusc harvesting. A well-known example is the area close to Porto Marghera in the Venice Lagoon, in Italy, where decades of discharging industrial wastes have led to high concentrations of several pollutants in the sediment (e.g. dioxins; PCBs; heavy metals). Despite the interdiction to harvest bivalves for human consumption in this area (Veneto regional regulations No 133/2018), however, illegal clam harvesting in Porto Marghera often occurs with major risks for consumers’ health.

For all the above reasons it is clear that developing tools that allow verification of bivalve origin as stated in the product label is increasingly important. A broad array of methodologies like fatty acids (FA) profiling, stable and unstable isotope and trace element fingerprinting, as well as several methods based on DNA analysis (DNA fingerprinting, microbial barcodes or profiles, among others), have been proposed as possible tools to trace the geographic origin and detect mislabelling [9–11]. These methods have variable performance depending on the geographic scale and mobility of the target species, and diverse feasibility in terms of time and cost of analysis. For instance, DNA analyses have been the most used tool for species identification (DNA barcoding) and the second most used to discriminate the geographic origin of bivalves (genetic traceability) [10]. This type of analysis is usually chosen for its relatively rapid and cost-effective results. However, the absence of barriers to the gene flow in species with highly vagile developmental stages may preclude genetic divergence between closely located populations (high connectivity), thus limiting the diagnostic power of genetic traceability [12]. FA profiles are among the most used and highly reliable approaches to discriminate the geographic origin of commercial bivalves as these species may modulate their FA profiles by several intrinsic (e.g. age, sex, reproductive cycle, and phylogeny) and extrinsic factors (e.g. diet, temperature, depth, and salinity) [13]. However, as previously mentioned for DNA analysis, recent studies have reported that natural seasonal and inter-annual FA variability may limit its accuracy when considering samples collected over multiple seasons/years [14].

A second major issue is that nearly all these methods have been validated using samples collected in different areas at a single time point (season, year). Even when a rigorous statistical analysis is implemented, site-of-origin discrimination accuracy is estimated based on a leave-one-out cross-validation. Such an approach is prone to overfitting, inflating the estimated accuracy. It also does not allow for the assessment of whether the discriminant function or the predictive model can generalise, i.e. correctly classifying never-seen-before samples. This is even more problematic when there are time dependencies among the data.

Microbiome profiling (MP) or 16S metabarcoding is an emerging alternative approach for unveiling geographical origin misrepresentations [15–17]. This method consists of the amplification and analysis of a fragment of the 16S rRNA gene from the microbial communities present in a given sample with the final aim to detect area-specific taxonomic composition profiles [18]. Recent studies have proven that although the microbial composition of molluscs is affected by environmental factors (e.g. temperature, salinity, chemical contamination), it is also characterized by a striking resilience to change; thus, making microbiota a suitable candidate for traceability [19, 20]. In fact, previous works employing this technique have reported its successful use to trace the geographic origin of commercially important wild [16, 21] and cultured species [22]. However, as mentioned above, molluscs grown in transition habitats such as lagoons and deltas need to undergo a process of depuration before commercialization. Such a process might significantly change their microbiota [15, 23] and ultimately impair our ability to discriminate between molluscs collected at differing locations.

The present study aimed at implementing MP in seafood traceability and addressing all the above-mentioned issues. First, samples were collected and processed (depurated) as is the case for commercial bivalve products. Second, the predictive model was based on two ML algorithms that are less prone to overfitting (linear bagging and random forests). The number of features used was limited to those taxonomic units (amplicon sequence variants, ASVs) showing significant abundance (> 5%), to reduce noise and limit the “large p small n” problem. Third, samples were collected across seasons to assess the effects of seasonality on prediction accuracy. Fourth, two comparable data sets were obtained from two different years, providing independent training/validation and test sets to correctly assess the generalization ability of the trained model. Briefly, 16S metabarcoding data for two tissues (gills (GI) and digestive glands (DG) of Manila clam) were obtained for samples collected at four different sites, representing the major clam farming areas in the North Adriatic Sea, across different seasons and years. A fifth site, the interdicted area of Porto Marghera was included to test the ability to classify illegally commercialized products harvested in potentially contaminated areas. Overall, high accuracy of prediction was observed, especially when identifying clams from the interdicted area. The predictive model was also robust to seasonal and inter-annual variation, showing significant generalization ability.

## Results

### Data summary

A total of 1000 clams were collected during field expeditions and pooled in 560 DNA-pool samples (280 per tissue). Raw DNA libraries were deposited at the NCBI repository under the BioProject access number: PRJNA1013079 [24]. After the quality-filter steps, a total of 15,491,493 reads were retained and rarefied at 5463 reads per sample. Finally, after the exclusion of rare ASVs (less than 5% of abundance), a number between 150 and 300 ASVs (depending on the comparison) were used to train ML algorithms.

### Predicting the origin of samples via ML

**Fig. 1:**
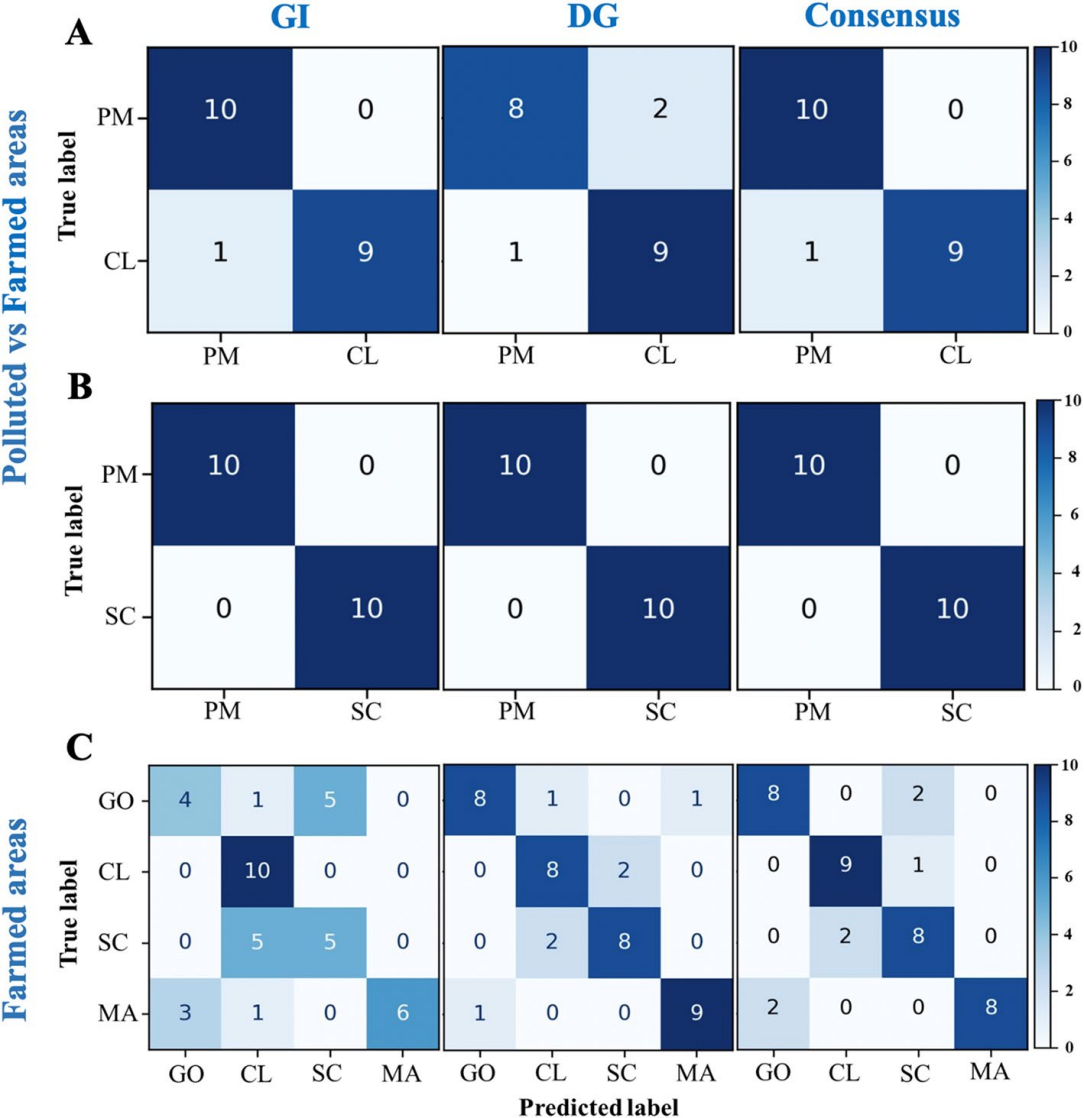
Confusion matrices showing the results of the ML predicted provenance (“Predicted label”) versus the real provenance (“True label”) for each of the tested samples by using gills (GI) only (left column), digestive gland (DG) only (middle column) or by combining GI and DG into a consensus prediction (right column). **A**, **B** Classification discriminating between the polluted site PM and the clean farming sites of CL (**A**) and SC (**B**). **C** Classification assessing the origin of samples among the four farming areas considered in the study. Colour scale is proportional to the number of samples that are assigned to a specific location. MA, Marano; PM, Porto Marghera; CL, Chioggia; SC, Scardovari; GO, Goro

In the comparison of Porto Marghera (PM) vs Chioggia (CL) (i.e. the polluted and farming sites within the Venice lagoon), the average precision in the prediction varied between 0.85 and 0.95 (Additional File 1: Table S1), with the GI tissue providing better results than the DG (Fig. 1). The consensus model allowed to reach an overall AUC of 0.95 with all samples from PM being correctly identified as PM while only 1 sample of CL was mislabelled (Fig. 1A). Binary classification was based on a large set of features (predictors), as the analysis of feature importance (Additional File 1: Table S1) showed that in the case of PM vs CL the presence-absence of 40 different ASVs for the GI and 50 ASV for the DG data explained 75% of classification importance. Similar evidence was observed for other classification tests by comparing PM vs other farming sites (Additional file 2: Fig. S1).

In the comparison of Porto Marghera (PM) vs Scardovari (SC) (i.e. the polluted site within the Venice lagoon and the farming site outside the Venice lagoon) ML was able to correctly identify all animals with 100% accuracy with no differences in performances among tissues considered (Fig. 1B).

**Fig. 2:**
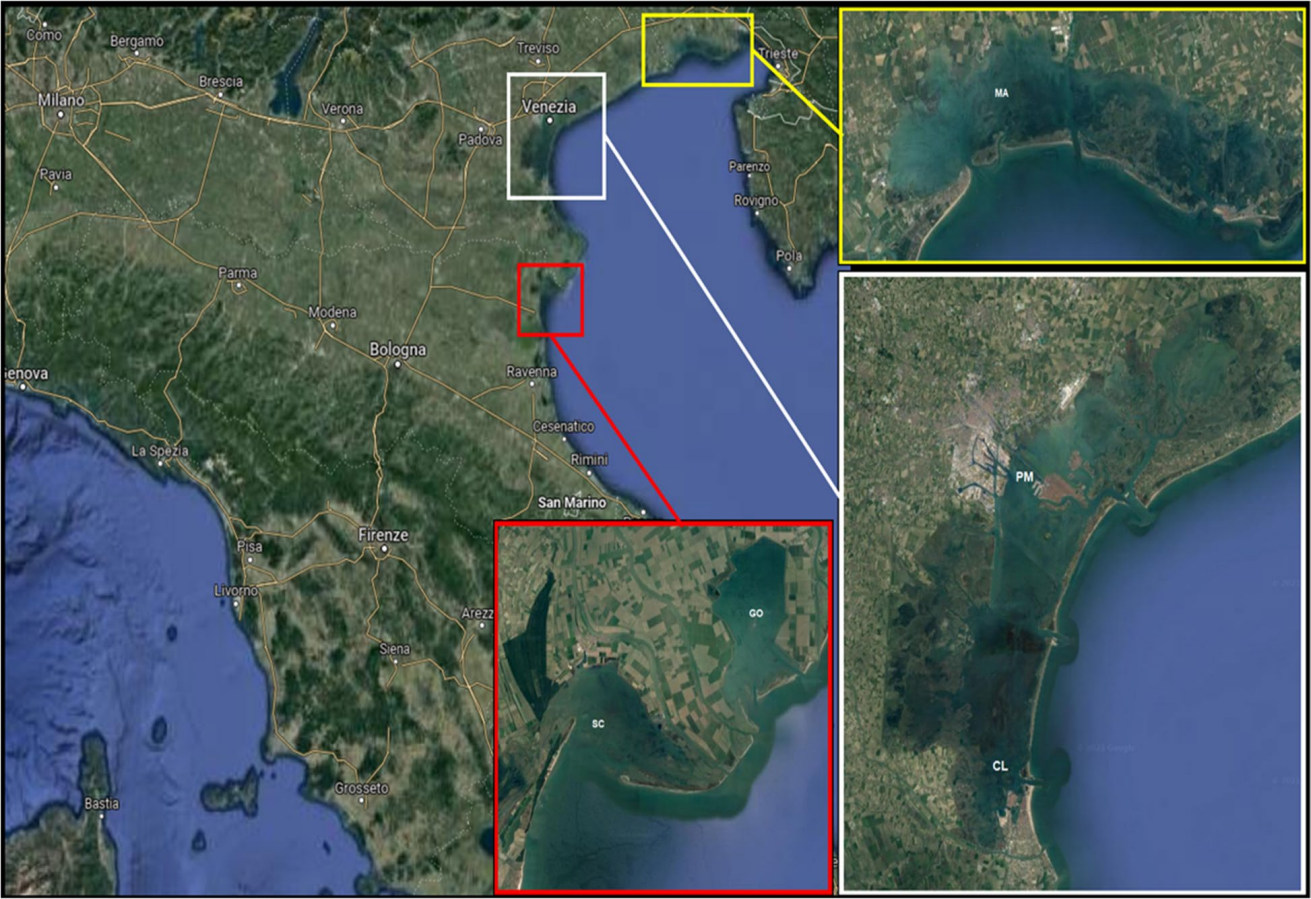
Map showing the sampling areas: two from the Venice lagoon, two from the Po river delta and one from the Marano and Grado lagoon. MA, Marano; PM, Porto Marghera; CL, Chioggia; SC, Scardovari; GO, Goro

The analysis between farmed areas was more challenging since accuracy ranged from 0.66 for GI to 0.83 for DG (Fig. 1C). Once again, better results were obtained with the consensus model that achieved an average accuracy of 0.84. Using the combined GI + DG data sets for every farming area, at least 8 out of 10 samples were correctly classified (Fig. 1C). Not surprisingly, the mislabelled samples from Goro (GO) were assigned to SC which is the closest site to GO in terms of geographic distance (Fig. 2).

## Discussion

Traceability, the possibility to track each step along the supply chain of any food product from its origin to the final consumer, is essential to ensure product quality and consumer safety. Traceability is particularly relevant for shellfish species. These organisms are filter-feeders that can bio-accumulate abiotic and biotic hazardous compounds if grown in areas subjected to chemical and/or biological contaminations, with potential risks for public health [2, 12, 25]. In fact, episodes of molluscs harvested from restricted areas and then sold in fish markets are still frequent and it can be quite difficult and cost- and time-consuming to detect such dangerous frauds. Thus, the creation of diagnostic tools able to accurately and easily discriminate the provenance of seafood, preventing the introduction of unsafe products in the market [12, 25] has long been sought for by health authorities. In addition, such tools could help producers and cooperatives to guarantee quality and environmental certifications, increasing consumer trust in specific brands and eco-labels [12, 26–28].

In a previous study, we showed the potential for ML-powered classification of MPs to discriminate clams from Porto Marghera compared to animals collected from farming sites located in the Venice lagoon [12]. However, in that preliminary study temporal replication was not extensive and sampling was limited to the Venice lagoon, excluding other important clam farming areas along the North Adriatic coast. In addition, a single tissue (digestive gland) was analysed and, most importantly, clams did not undergo the depuration process required by legislation, thus ignoring the potentially altering effects of this treatment on the animal microbiota. It has in fact been demonstrated that depuration may significantly influence the bivalve microbial community [15]. In the present work, we carried out an entirely new sampling campaign, with extensive temporal replication and broad geographic representation. All samples underwent the standard depuration process, which constitutes a key step forward. In fact, we have successfully demonstrated that, despite the depuration process, a distinctive “signature” is still detectable in the clam MP. As already mentioned, existing evidence suggested that the composition of host-associated microbiota was relatively stable and distinct from the microbial communities present in the water and the sediment [20]. This is likely explained by the interactions between host and microbiota, which select and maintain distinctive bacterial taxa associated to specific organs and tissues [29].

The dynamics of host colonization and evolution of host-microbiota associations, however, remain to be better elucidated in bivalves, and such knowledge might be highly relevant to more accurately understand the potential and the limitations of MP analysis in traceability. A first attempt in shading light on the dynamics of clam biology and its associated microbiota has been taken recently by Milan et al. [19] and by Cecchetto et al. [30] by performing long-term monitoring campaigns on clams farmed in four sites of the Venice lagoon. Our works revealed that clams undergo biotic and abiotic stressors in a site-specific way, with locations in close proximity to the inlet of the lagoon experiencing more frequent salinities and/or temperatures beyond the optimum range of the species. Further, we showed how environmental gradients, such as salinity and water residence time, influenced the overall gene expression pattern of clams, the beta diversity of microbial communities in the host, and putatively translated into differences in clam’s growth, condition index (an index of general well-being of the animal) and mortality. Interestingly, we found an over-representation of the genera *Arcobacter* and *Vibrio* (both described as opportunistic pathogens) at the end of the summer in sites closer to the inlet and thus potentially subject to a higher abiotic stress. Unfortunately, we did not have multi-parametric probes in place when we performed all sampling activities for the current work, thus hampering the possibility to integrate environmental data in our ML-based traceability system.

Another key outcome of the present study is the highly significant accuracy (> 95%) in discriminating clams collected in the prohibited area (PM) from those harvested in the nearby farming sites within the Venice lagoon and even more accurately (100% recovery) when samples originated from a geographically close, but distinct lagoon (SC). Similar results were obtained for pairwise comparisons between PM and the two other farming sites. We believe this is a significant proof-of-concept that ML-empowered analysis of MPs could be used to independently verify the origin of bivalves suspected of being illegally collected in areas that are interdicted for various reasons, as in the case of sites showing high levels of chemical and/or microbiological contamination. The specific environment present at contaminated sites might shift the composition of microbial communities, making MPs more easily identifiable. A second potential factor that might boost classification accuracy is the fact that clams in PM are generally undisturbed after settlement, therefore early on during their life history, making them long-term residents in the same area and allowing them to be associated with a highly distinctive microbiome. This is not the case of clams collected in authorized farming sites as it is a common practice to use already metamorphosed juvenile individuals to seed on-growing sites. As mentioned, little is known about the dynamics of the establishment and maintenance of clam-associated microbiota, although the early life history phases seem to be crucial in determining microbiome composition in adult animals as observed in aquatic model species [31]. The frequent practice of seeding farming sites with juveniles from different areas or with spat produced by captive reproduction in bivalve hatcheries might explain the lower, though significant accuracy (*k*-score 0.76 compared to the expected 0 by random chance) in classifying the four most important Italian production areas. In fact, juveniles from natural nursery areas from the Po river delta (Scardovari and Goro; SC and GO) have been reported to be used to support farming sites in the Venice and Marano lagoons (CL and MA), where natural recruitment has been extremely limited in recent years. It should also be noted that multi-class discrimination cases are generally more problematic than binary classification, which might add to the problem of translocation of juvenile clams.

In the present study, ML-based classification provided excellent or very good accuracy. We believe that several factors contributed to such a positive outcome. First, presence-absence data were used, enforcing a conservative threshold (> 5%) for considering a specific ASV as present. This approach reduces the potential noise linked to fluctuating abundance caused by technical and/or biological factors. Second, ASVs were used as features for classification, which means that discrimination is based on the presence of “unique” bacterial strains/species in a specific site. Third, reducing the number of features likely limited the risk of overfitting, which is generally high for ML-based classification methods. Fourth, a bootstrap aggregating (bagging) approach was implemented, which is expected to further reduce the problem of overfitting. Fifth, two different classification models were tested (linear bagging and random forests), and the one providing the best classification performance was used in the validation step. Last, and most important, as already mentioned, the set of samples used to train the classification algorithm was completely independent from the validation set. To the best of our knowledge, even for studies in which the classification tool (either using traditional statistical approaches or ML methods) was validated, the validation was performed as a leave-one-out cross-validation approach. This is known to greatly inflate the estimated accuracy and the generalization ability of the model. Our work shows that MP analysis using properly selected and trained ML models is robust to seasonal variation and truly able to predict the origin in never-seen-before samples (collected in a different year). Indeed, reproducibility across temporal replicates is certainly one of the most relevant features for any traceability tool, but unfortunately is greatly overlooked.

Overall, the present study provides compelling evidence that traceability tools based on ML-enabled analysis of host-associated microbiota might be a powerful weapon in the war against illegal seafood trading. At the moment we have tested our ML method focusing on a relatively “short” geographic distances (even if we have included all major Manila clam farming sites of Europe in terms of production [32]) because it is more challenging to discriminate the provenance of samples on small geographic distances rather than on broader distances, as demonstrated by Mamede et al. [33]. However, the decreasing costs of DNA sequencing will also make it possible to create much larger training data sets, with broader geographic coverage and temporal replicates. This, in turn, should greatly improve classification accuracy and limit the “large p small n” problem, where p is the number of features/predictors used and n is the number of cases. The rapid and continuous decline of cost for DNA sequencing will make these tools increasingly affordable and the cost is already on par or even lower than for other methods such as FA profiling and trace elements analysis. Furthermore, DNA 16S rRNA metabarcoding data easily comply with the FAIR principles (Findability, Accessibility, Interoperability, and Reuse), as protocols and public data repositories are well established for this type of data, although further efforts are needed especially toward the standardization of DNA extraction methods and the harmonization of the 16S rRNA gene region to be used are needed. Based on our results and other available evidence, we believe that the relative stability of host-associated microbiota likely allows high traceability accuracy for sessile species (e.g. bivalves, macroalgae). For species that are highly mobile, such stability might trace the area where the animal/plant was located during the early life stages, but be less precise in tracking more recent movements.

Another potential limitation is the time required for data production and analysis. Currently, the limiting step is DNA sequencing, which is based on Illumina technology and requires a couple of weeks to obtain DNA sequence data. However, the advent of third-generation sequencing technologies is making it possible to greatly speed up the process and in the case of Nanopore 16S barcoding kit, also to make it easily portable if implemented on a MinION instrument. In addition, long-read sequencing provides full length sequence information for the 16S rRNA gene, overcoming the previously mentioned problem of finding a consensus for the gene fragment to be sequenced and, at the same time, allowing for increased taxonomic resolution, with a higher likelihood to identify site-specific ASVs. We expect that in the near future, it will be possible to carry out rapid, in-the-field, 16S rRNA metabarcoding analyses. In fact, rapid DNA extraction protocols suitable for long-read sequencing are already becoming available [34], the array of technological solutions for portable, miniaturized PCR thermal cyclers is rapidly expanding [35], and the use of pre-trained ML-based algorithms for 16S data analysis using cloud computing has already been reported [36]. Furthermore, ML performance usually increases when the data size grows. Thus, we may expect significant improvement when more samples are collected and used to train the models. However, the successful implementation and improvement of the model will strongly depend on a close collaboration with regulatory bodies and industry stakeholders which will also favour its adoption and further extend its usage.

## Conclusions

In conclusion, the present study demonstrates that ML-enabled analysis of host-associated microbiota is already a key instrument complementing the toolbox for seafood traceability, showing the importance of addressing the most relevant steps to ensure classification accuracy. Looking ahead, we expect that highly portable and interoperable diagnostic tools based on the approach proposed here will become available, making prevention of illegal seafood trade rapid and affordable.

## Methods

### Samples collection, DNA extraction and sequencing

Commercially harvestable adults of Manila clam *Ruditapes philippinarum* were collected during four sampling campaigns conducted in July 2018 (S18), January 2019 (W19), July 2019 (S19) and January 2020 (W20) along the Venice lagoon and neighbouring areas (Fig. 2). During each sampling expedition, around 100 clams were collected from four farming areas (i.e. Marano Lagunare (MA); Chioggia (CL); Scardovari (SC); Goro (GO)) and one polluted site (i.e. Porto Marghera (PM)) by a mechanical rake and following official regulations for bivalve commercialisation. Landed animals were kept in a depuration centre for 16 h where they were kept in open flow-through systems that continuously receive natural sea water that is mechanically, biologically and chemically (i.e. UV) filtered. After this step, animals were brought to the laboratory where the entire gills (GI) and digestive gland (DG) were dissected from each clam using sterilized scalpels. Tissue samples were immediately transferred to 1.5-ml microcentrifuge tubes containing molecular grade ethanol (90%). Samples were then refrigerated at 4 ℃ until further analysis. DNA was extracted from pooled tissues obtained by pooling 10 GI or DG tissue pieces of similar weight from clams collected from the same expedition and area. DNA was extracted and purified using a DNA Power Soil kit (QIAGEN, Hilden, Germany) following the manufacturer’s instructions with an additional step of Proteinase K to improve cell lysis. DNA integrity was verified using agarose gel electrophoresis (1%), while its quantity was estimated by NanoDrop ND1000. DNA aliquots were sent to BMR Genomics (Padua, Italy) where a fragment of the 16S rDNA spanning the V3-V4 regions was PCR-amplified and sequenced using MiSeq 2 × 300 pair-end (PE) sequencing technology.

### 16S rRNA libraries preparation

Raw reads were processed and analysed using Quantitative Insights into Microbial Ecology 2 v. 2019.1 (QIIME 2) [37]. Primer sequences were removed using cutadapt [38]. DADA2 [39] was used to filter low-quality sequences and to merge forward and reverse libraries to obtain high-quality representative sequences. The same program was also used to remove chimeric reads from the final sequence dataset. Representative sequences were aligned using MAFFT software [40] and classified using the Python library Scikit-Learn [41]. Taxa assignment was carried out using the v.142 SILVA database trained for V3-V4 regions. Finally, in order to standardize reads libraries to a common sampling depth, reads were rarefied by randomly subsampling by the minimum number of reads. These QIIME 2 outputs, including the abundance feature table and taxonomy, were used in the training and testing for machine learning (ML) based classification.

### Machine learning procedures

Using ML, two classification problems were tested: (*i*) the possibility of discriminating between clams harvested in authorized farming areas and those illegally collected in the interdicted polluted; (*ii*) the possibility of tracing the origin of clams grown in the four most important farming sites of the north Adriatic Sea, as each is these areas carries its own commercial brand (e.g. “Vongola verace di Scardovari”). In the first case, we trained and tested the ML-based tool in a two-class classification problem, i.e. to discriminate between samples from the interdicted area (PM) and the geographically closest farming site, Chioggia (CL) (Fig. 2). Likewise, we tested the comparison between PM and the most important farming area, Scardovari (SC). The second scenario consisted in a multiple class classification problem, where we tested the ability of the ML algorithm to discriminate the provenance of clams by including all farming sites together (i.e. GO; CL; SC; MA).

### Preparation of input data

For all case studies, input data were filtered to remove ASVs with low counts, to reduce noise and to select a smaller number of features (predictors). In total for each case study, 8 input files were generated, one for each combination of tissue (i.e. DG and GI) and sampling time (i.e. S18, S19, W19, W20). More in detail, the ASVs table was initially imported in R/v4.2.1 (R Core Team, 2014) and filtered in order to keep only locations that were involved in the relevant comparison. Then, for each tissue and season (i.e. Summer or Winter) the ASVs table was split between the first and second year of sampling and was filtered to keep only ASVs with an abundance higher than 5% in at least one of the samples, in both years. Then the filtered ASV tables were converted to binary matrices (i.e. presence/absence), and the taxonomy file was updated to keep only ASVs that were present in the binary matrices. The R code used to generate the input files can be found on GitHub at the following link [42]: https://github.com/GEMMA-BCA/Machine-Learning-assisted-Microbiome-analysis.

### Identifying clams from the interdicted area

Binary matrices from ASV tables were imported in Jupiter notebook v6.4.12 (https://jupyter.org). The library scikit-learn [41] was loaded in python/v3.9.13 and used to train a random forest (RF) classifier with the function “RandomForestClassifier”. Initially, the RF model was cross-validated (i.e. where the training set was split into k-folds, k-1 folds were used for training and the resulting model was validated on the remaining part of the data) to define the best parameters (i.e. the number of estimators “n_estimators”, ranging from 12 to 18, and the max depth of trees “max_depth”, ranging from 1500 to 2000) and the model with the highest Cohen kappa score was chosen. Cross-validation was performed on second-year data only (i.e. S19 + W20 together) and a separate RF model for each tissue was built and cross-validated independently. After cross-validation, data from the second year was fitted on the optimized RF model, and then this was used to predict data on novel, never-seen-before data from the first year (i.e. S18 + W19) on each tissue separately.

The performance of the model in predicting the provenance of clams sampled in the first year was assessed separately for each tissue by using the Area Under the Curve of a Receiver Operating Characteristic AUC-ROC with the function “roc_auc_score” and by means of confusion matrices. For each model the most important features were obtained using the function “feature_importances”. In addition, we assessed if a consensus model, derived by combining the separate predictions from the two tissues, would improve the performances over the single-tissue models. For this purpose, we obtained the predicted class probabilities of each input sample from both tissues with the function “predict_proba”; then for each sample these probabilities were summed, and the class having the highest sum was deemed as the predicted class of this consensus model. The performance of the consensus model was evaluated by using AUC-ROC score and confusion matrices, as previously described.

### Tracing farming sites

For the multiple class problem, instead of a RF classifier, a multinomial logistic regression was set up with the function “LogisticRegression” of the python sklearn library and the following options: “multi_class = ’multinomial’, solver = ’lbfgs’, penalty = ’l2’, class_weight = ’balanced’, fit_intercept = False, C = 1”. To achieve a better final prediction, this “base” model was coupled with bootstrap aggregation by using the ensemble meta-estimator bagging classifier (with the function “BaggingClassifier”). Cross-validation was used to define the best parameters of the bagging classifier (i.e. the number of estimators “n_estimators”, ranging from 500 to 1500, and the maximum number of features to train each base estimator “max_features”, ranging from 100 to 300) and the model with the highest Cohen kappa score was chosen. Cross-validation was performed on second-year data only (i.e. S19 + W20 together) and a separate model for each tissue was built and cross-validated independently. After cross-validation, data from the second year was fitted on the optimised bagging classifier model, and then this was used to predict data on novel, never-seen-before data from the first year (i.e. S18 + W19) on each tissue separately.

The performance of the model in predicting the provenance of clams was assessed as described in the previous section. Similarly, the assessment of the performances of a consensus model derived by combining the separate predictions from the two tissues was performed as described above. Finally, for each model, the most relevant features were extracted using a custom python function and, for each farming site, the top 15 features were plotted using the R package ComplexHeatmap/v2.14.0 [43].

## Supplementary Information

Additional file 1: Table S1. Precision, recall, f1-score and support obtained in gills (GI) and digestive gland (DG) obtained in comparing Porto Marghera versus Chioggia (PM vs CL), Porto MArghera  versus Scardovari (PM vs SC), farmin sites. Feature importance in gills and digestive are also reported.

Additional file 2: Figure S1. Confusion matrices showing the results of the ML predicted provenance (“Predicted label”) versus the real provenance (“True label”) for each of the tested samples by using gills (GI) only (left column), digestive gland (DG) only (middle column) or by combining GI and DG into a consensus prediction (right column). **A**) Classification discriminating between the polluted site PM and the clean farming sites of MA. **B**) Classification discriminating between the polluted site PM and the clean farming sites of GO.

## Data Availability

Raw DNA libraries were deposited at the NCBI repository under the BioProject access number: PRJNA1013079 [24]. The R code used to generate the input files can be found on GitHub at the following link [42]: https://github.com/GEMMA-BCA/Machine-Learning-assisted-Microbiome-analysis.

## Abbreviations

MP: Microbiome profiling
ML: Machine learning
GI: Gills
DG: Digestive gland
ASVs: Amplicon sequence variants
PCBs: Polychlorobiphenyls
FA: Fatty acids
MP: Microbiome profiling
MA: Marano
CL: Chioggia
SC: Scardovari
GO: Goro
PM: Porto Marghera
RF: Random forest
