# Supplementary figures and images for: Preventing illegal seafood trade using machine-learning assisted microbiome analysis
Source: BMC Biol. 2024 Sep 11;22:202. doi: 10.1186/s12915-024-02005-w (PMC11389296; doi:10.1186/s12915-024-02005-w)

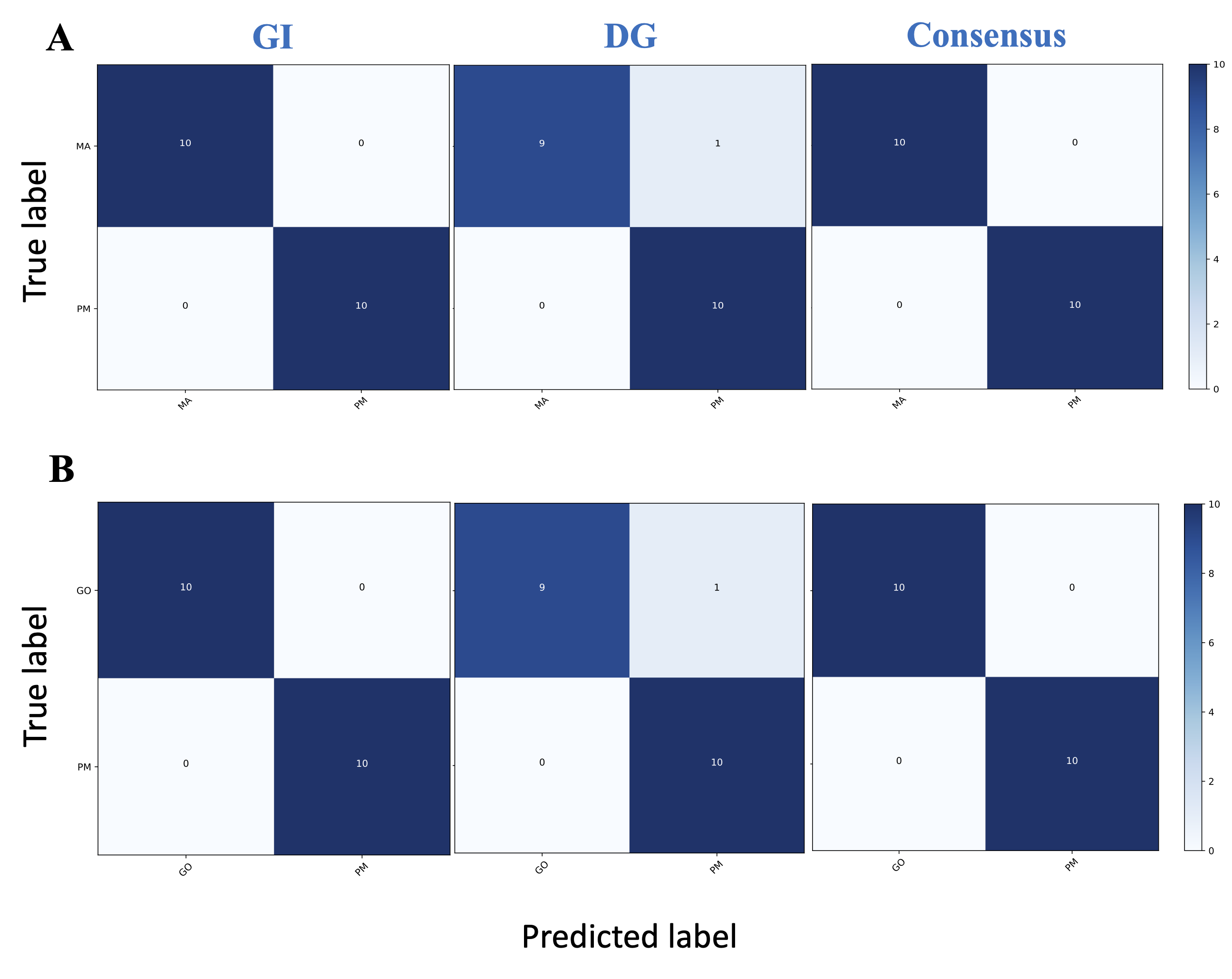

Supplement: Supplementary file 2 — Additional file 2: Figure S1. Confusion matrices showing the results of the ML predicted provenance (“Predicted label”) versus the real provenance (“True label”) for each of the tested samples by using gills (GI) only (left column), digestive gland (DG) only (middle column) or by combining GI and DG into a consensus prediction (right column). A) Classification discriminating between the polluted site PM and the clean farming sites of MA. B) Classification discriminating between the polluted site PM and the clean farming sites of GO. [file 12915_2024_2005_MOESM2_ESM.tiff]
